# Giant Arachnoid Granulation Associated with Anomalous Draining Vein: A Case Report

**DOI:** 10.7759/cureus.1065

**Published:** 2017-03-01

**Authors:** Randle Umeh, Rod J Oskouian, Marios Loukas, R. Shane Tubbs

**Affiliations:** 1 Department of Anatomical Sciences, St. George's University School of Medicine, Grenada, West Indies; 2 Swedish Neuroscience Institute; 3 Department of Anatomical Sciences, St. George's University School of Medicine, Grenada, West Indies; 4 Neurosurgery, Seattle Science Foundation

**Keywords:** anatomy, intracranial, dural venous sinus, variation, arachnoid matter

## Abstract

Giant arachnoid granulations (AG) can mimic intracranial lesions. Knowledge of these structures can help avoid misdiagnosis when interpreting imaging. Here, we report a child who presented with a mass within the superior sagittal sinus and an anomalous draining vein. Herein, the diagnosis of a giant AG was made. Clinicians who view or interpret imaging of the head should be aware of these anatomical variants and though when very large, apparently, do not necessarily result in pathology. Based on our case report, giant AG might also demonstrate anomalous draining veins.

## Introduction

Arachnoid granulations (AG), first described in 1705 by Italian anatomist Antonio Pacchioni, are cerebrospinal fluid-filled protrusions of the arachnoid mater that extend from the subarachnoid space into the venous system through apertures in the dura mater [1-2]. AG are lined by meningothelial cells and function in reabsorption, filtration, and drainage of CSF from the subarachnoid space into the venous circulation [1-3]. These hypertrophied arachnoid villi become distended through increasing CSF pressure and volume, typically ranging in size from 2–8 mm allowing for gross visualization [3]. Seen as early as at four years of age, AG number and size increases with age [3-4]. Herein, we describe a case of a giant AG associated with an anomalous draining vein. Informed consent was obtained from the patient for this study.

## Case presentation

**Figure 1:**
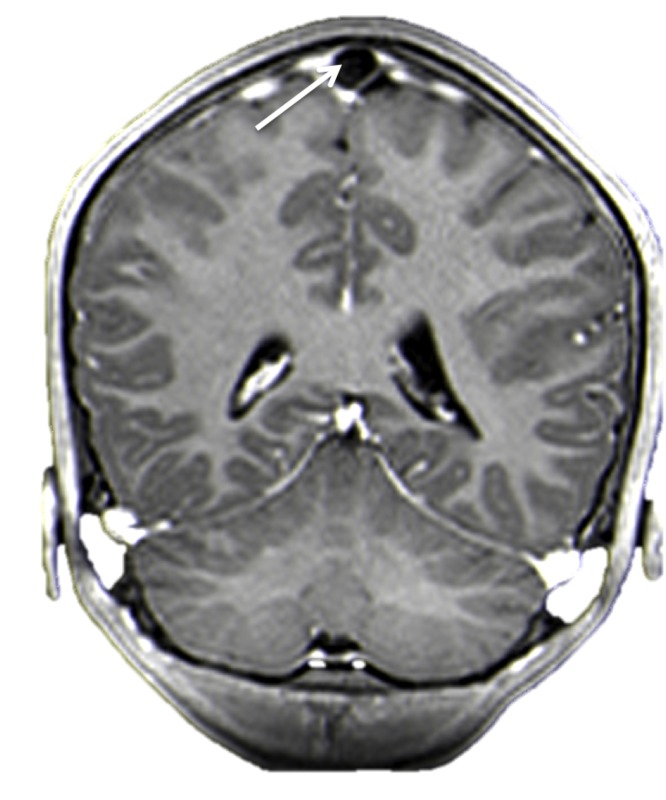
Contrasted coronal MRI of the head. Note the large hypodense area (arrow) within the superior sagittal sinus. This was diagnosed as a giant AG.

**Figure 2:**
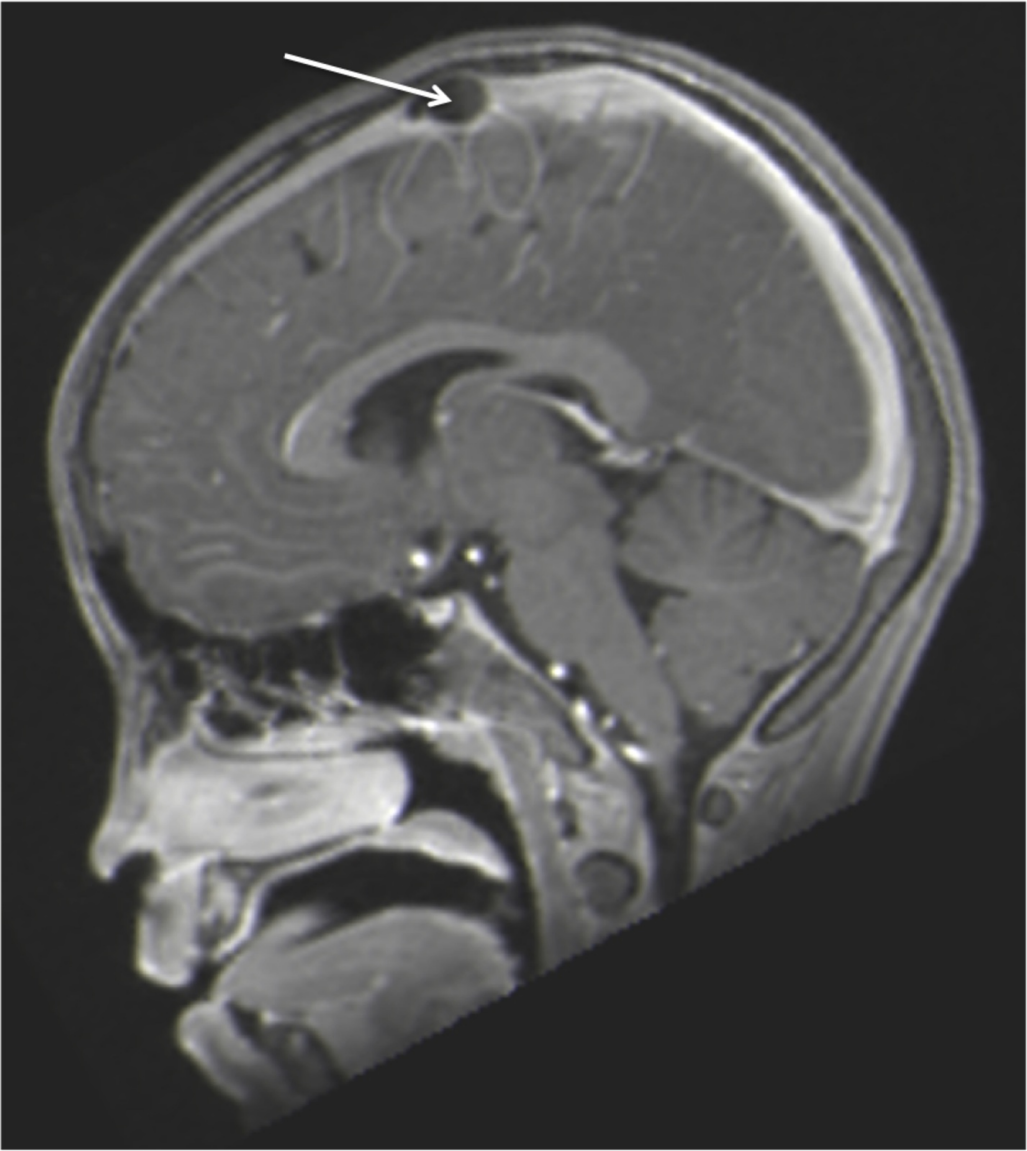
Sagittal contrasted MRI noting large mass (arrow) in the superior sagittal sinus.

**Figure 3:**
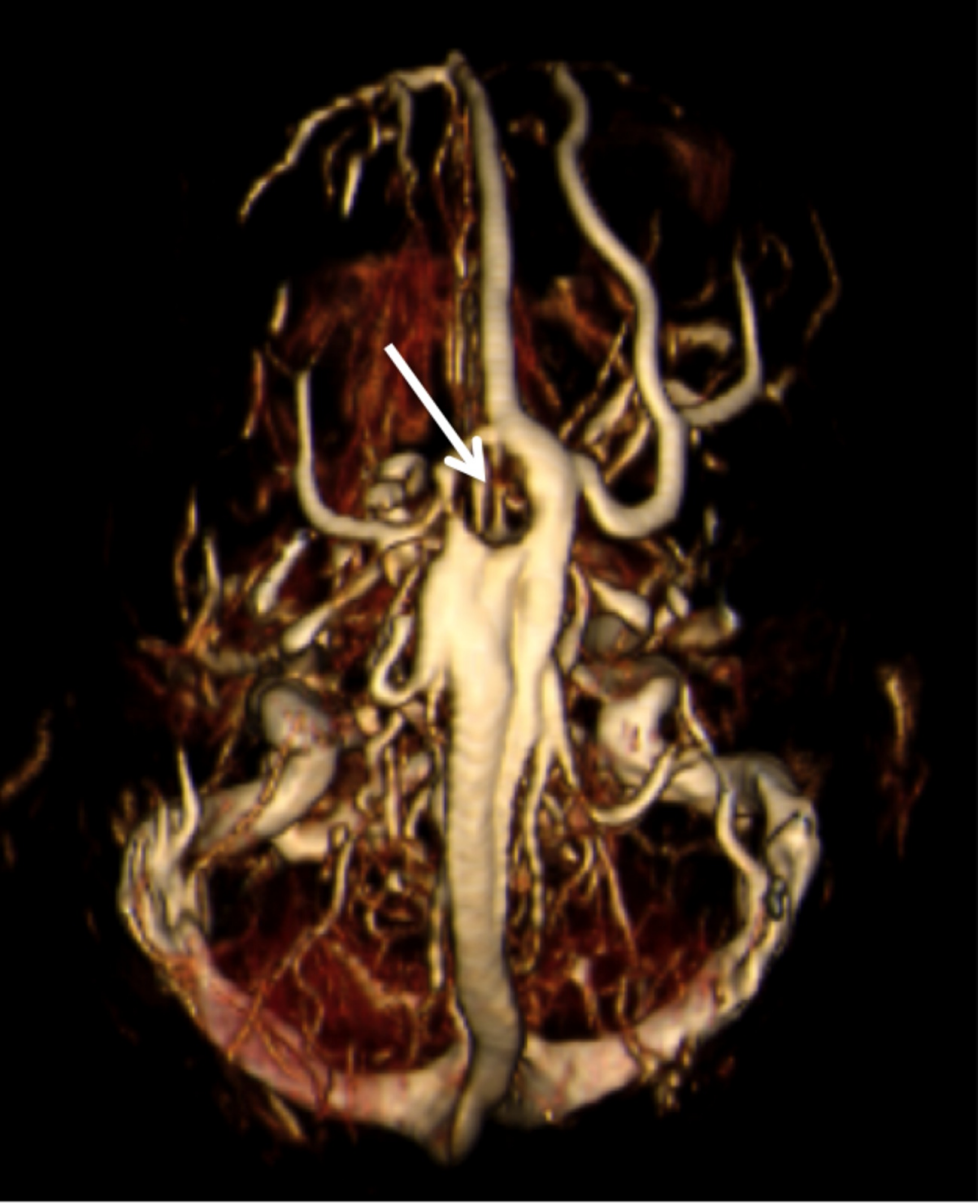
T2-weighted coronal MRI of the brain noting giant AG (arrow).

**Figure 4:**
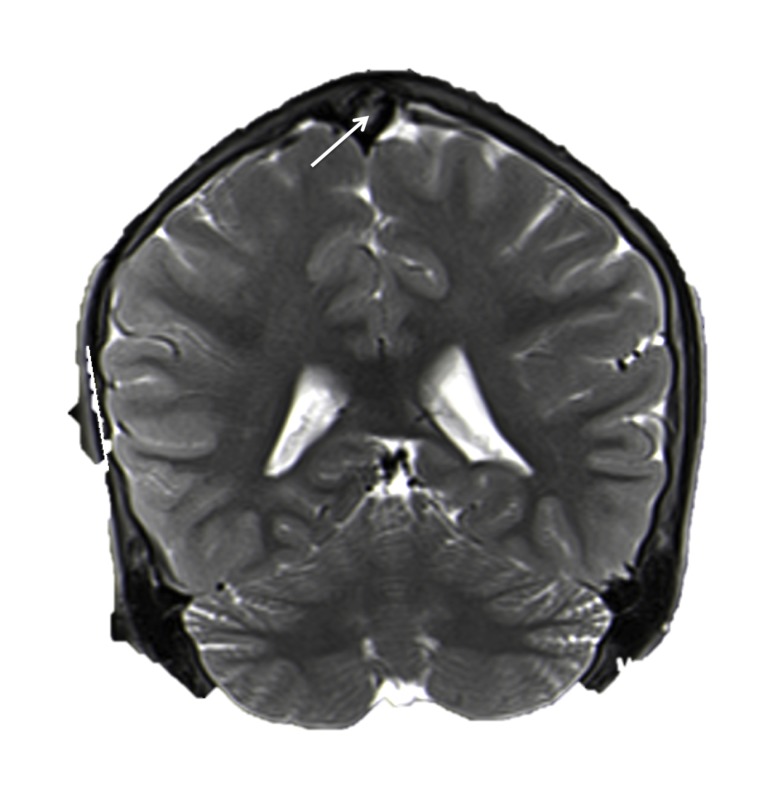
MRV of the head noting the large AG causing a defect (arrow) in the superior sagittal sinus. Also, note the left-sided anomalous draining vein running parallel to the superior sagittal sinus.

A previously healthy 16-year-old female presented with a single syncopal episode while at school. She also complained of recent headaches that were worse upon wakening. The headaches had begun two months earlier and were treated with over the counter non-steroidal anti-inflammatory medicines. She was on no medications and had a surgical history of tonsillectomy at the age of six years. A magnetic resonance imaging (MRI) scan of the head was obtained to evaluate intracranial anatomy. This revealed a large mass (1.2 x 2.3 cm) in the midline and seemingly, within the superior sagittal sinus (Figures 1-3). A magnetic resonance venography (MRV) scan (Figure 4) demonstrated a large punched out defect in the superior sagittal sinus at the junction of its posterior two thirds and anterior one third. An abnormal and large cortical vein was noted on the left side that traveled parallel to the superior sagittal sinus. The cortical vein drained the left frontal region up to the frontal pole and at its drainage into the superior sagittal sinus, made an unusual loop before entry. The superior sagittal sinus just posterior to the midline defect was slightly enlarged. The large defect was diagnosed as an extremely enlarged AG. There were no other intracranial variants appreciated. Follow-up imaging at one year demonstrated no change in the size of the AG, and at that time, the patient’s headaches had resolved and no further syncopal episodes had been experienced.

## Discussion

There is no consensus in the literature as to when an AG is termed 'giant'; however, those that increase in size greater than 1 cm or cause local dilatation and filling defects by filling the lumen of a dural venous sinus are considered enlarged [1-2, 4]. Giant AG have commonly been discovered as incidental findings in the transverse and posterior superior sagittal sinus. They have rarely caused any symptoms related to an increase in intracranial pressure from partial venous sinus occlusion secondarily causing venous hypertension [4]. When patients do present with symptoms, headaches are the most common complaint [2]. Expansion of the inner table of the skull most commonly around the midline can also occur if these extensions from the arachnoid mater grow to sufficient size [1].

A large number of AG have been described in the posterior and middle cranial fossae [4-6]. They have also been noted in the olfactory mucosa and cranial nerve sheaths [7-8]. On imaging, normal and giant AG have typical characteristics [2]. Skull radiography will show AG as visible radiolucent zones or show the extensions causing an impression on the inner table of the calvaria [2]. On brain computed tomography (CT) and MRI, AG are seen as hypodense or isodense to brain parenchyma [8]. Giant AG within the dorsal superior sagittal sinus can be misdiagnosed as a dural sinus thrombosis [4,9] with MRI being a useful tool in differentiating the two [4].

## Conclusions

Giant AG can mimic intracranial lesions. Clinicians who view or interpret imaging of the head should be aware of these anatomical variants, and though when very large, apparently, do not necessarily result in pathology. Based on our case report, giant AG might also demonstrate anomalous draining veins.

## References

[1] Kan P, Stevens EA, Couldwell WT (2006). Incidental giant arachnoid granulation. Am J Neuroradiol.

[2] Keyzer BD, Bamps S, Calenbergh FV, Demaerel P, Wilms G (2017). Giant arachnoid granulations mimicking pathology: a report of three cases. Neuroradiol J.

[3] Trimble CR, Harnsberger HR, Castillo M, Brant-Zawadzki M, Osborn AG (2010). "Giant" arachnoid granulations just like CSF? NOT!!. Am J Neuroradiol.

[4] Choi HJ, Cho CW, Kim YS, Cha JH (2017). Giant arachnoid granulation misdiagnosed as transvers sinus thrombosis. J Korean Neurosurg Soc.

[5] Roche J, Warner D (1996). Arachnoid granulations in the transverse and sigmoid sinuses: CT, MR, and MR angiographic appearance of a normal anatomic variation. Am J Neuroradiol.

[6] Miyajima M, Arai H (2017). Evaluation of the production and absorption of cerebrospinal fluid. Neurol Med Chir (Tokyo).

[7] Tsutsumi S, Ogino I, Miyajima M, Nakamura M, Yasumoto Y, Arai H, Ito M (2014). Cranial arachnoid protrusions and contiguous diploic veins in CSF drainage. Am J Neuroradiol.

[8] Chen F, Deng XF, Liu B, Zou LN, Wang DB, Han H (2011). Arachnoid granulations of middle cranial fossa: a population study between cadaveric dissection and in vivo computed tomography examination. Surg Radiol Anat.

[9] Leach JL, Meyer K, Jones BV, Tomsick TA (2008). Large arachnoid granulation involving the dorsal superior sagittal sinus: findings on MR imaging and MR venography. Am J Neuroradiol.

